# Enhancing the systems productivity and water use efficiency through coordinated soil water sharing and compensation in strip-intercropping

**DOI:** 10.1038/s41598-018-28612-6

**Published:** 2018-07-12

**Authors:** Guodong Chen, Xuefu Kong, Yantai Gan, Renzhi Zhang, Fuxue Feng, Aizhong Yu, Cai Zhao, Sumei Wan, Qiang Chai

**Affiliations:** 1 Gansu Provincial Key Lab of AridLand Crop Science, Gansu Agricultural University, Lanzhou, 730070 China; 2 College of Agronomy, Gansu Agricultural University, Lanzhou, 730070 China; 3 College of Plant Sciences, Tarim University, Xinjiang, 843300 China; 4 Agriculture and Agri-Food Canada, Research and Development Centre, Swift Current, Saskatchewan Canada

## Abstract

In arid areas, water shortage is threating agricultural sustainability, and strip-intercropping may serve as a strategy to alleviate the challenge. Here we show that strip-intercropping enhances the spatial distributions of soil water across the 0–110 cm rooting zones, improves the coordination of soil water sharing during the co-growth period, and provides compensatory effect for available soil water. In a three-year (2009–2011) experiment, shorter-season pea (*Pisum sativum* L.) was sown in alternate strips with longer-season maize (*Zea mays* L.) without or with an artificially-inserted root barrier (a solid plastic sheet) between the strips. The intercropped pea used soil water mostly in the top 20-cm layers, whereas maize plants were able to absorb water from deeper-layers of the neighboring pea strips. After pea harvest, the intercropped maize obtained compensatory soil water from the pea strips. The pea-maize intercropping without the root barrier increased grain yield by 25% and enhanced water use efficiency by 24% compared with the intercropping with the root barrier. The improvement in crop yield and water use efficiency was partly attributable to the coordinated soil water sharing between the inter-strips and the compensatory effect from the early-maturing pea to the late-maturing maize.

## Introduction

Millions of people in populated countries, such as China and India, live on small-scaled, self-sufficient family farms^1^. Many of those farms are facing significant challenge that continuously producing sufficient quantities of grain to meet the needs of growing population while doing so with limited available resources^2^ and under variable climatic conditions^3^. In the arid to semiarid northwestern China, for example, annual precipitation is between 50 and 170 mm, while annual evaporation is greater than 2100 mm^4^. Historically, agriculture relies on belowground water for irrigation^4^, but the depth of water table has declined substantially in recent years due to climate change and over-exploration of underground water resources^5^. Water shortage is threatening crop production and agricultural sustainability^6^. An added pressure is the severe competition for water resources between agricultural sector and the fast-developing urbanization^7,8^.

Various means have been used to save water in agriculture, including the adoption of regulated deficit irrigation in crop production^4^, the use of innovative water-saving practices^9^, and the enforcement of bylaws and policies in water resource management^4^. One of the most effective approaches to improve water use efficiency (WUE, i.e., grain yield per unit of water supplied) in the production of field crops is the use of strip-intercropping, a cropping practice where an early-sown, cool-season crop is relay-planted with a late-sown, warm-season crop in strips on the same field^10^. Intercropping has been reported to enhance soil water conservation and reduce run-off^11^, increase the use of available soil water^12,13^, and improve crop yield for the entire systems^11^ and the yield per unit of water supplied^14–16^, as well as the yield of crops grown the following year in the rotation^11^. In arid and semiarid areas, use of intercropping in combination with regulated deficit irrigation^4^ or crop straw mulching^17^, can enhance WUE significantly^9,12^.

However, the mechanism causing the increased WUE in strip-intercropping is poorly understood. A number of studies have shown that increased WUE is due to the increased total yield per unit of water supplies. Aboveground interspecies interactions help improve stereo-structure avail in light capture between the two contrasting crops^18–20^ and enhance the light environment such as red to far-red ratio and the photosynthetically active radiation transmittance^21^. In the dry areas with high soil evaporation, it is most likely that belowground interspecies interaction for soil water sharing between the intercrops or soil water transformation in the rooting zones may occur^22–24^. However, there is a lack of quantitative determination in regard to how much soil water may be shared between the two intercrops during their co-growth period. It is unknown whether the two intercrops actually compete for soil water or may provide a compensatory effect by one intercrop to the other. The current knowledge is limited regarding how soil water sharing or compensation many actually happen under different levels of soil water availabilities.

**Figure 1:**
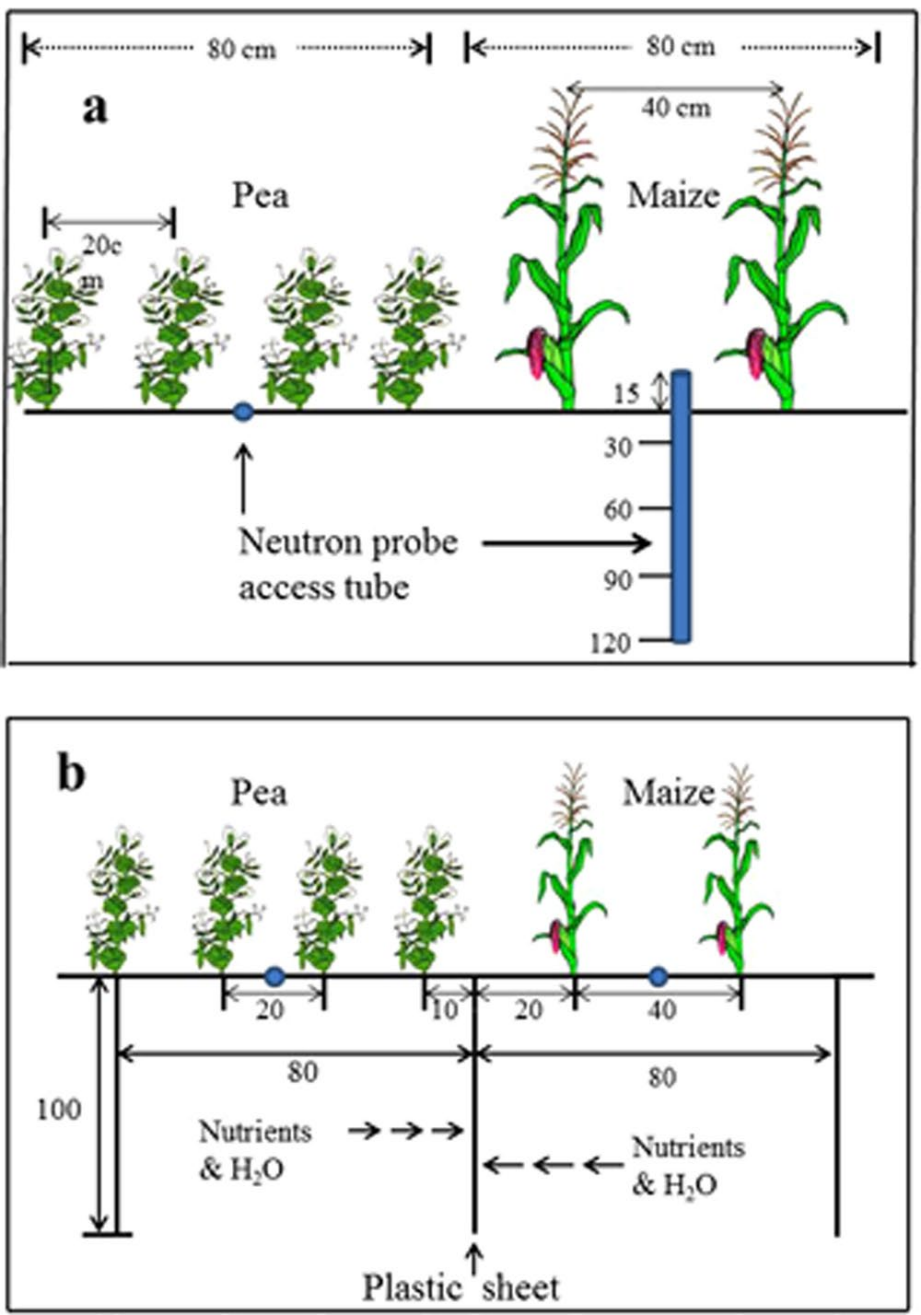
Field layout of pea planted with maize in alternate strips. (**a**) pea-maize strip-intercropping with no root barrier between the two strips, and (**b**) pea-maize intercropping with a solid plastic sheet inserted between maize and pea strips to the depth of 1.0 m prior to sowing. The blue dots indicate the positions where the soil moisture measurements were taken, and the blue tube indicates the depth of soil moisture measured.

Therefore, the objectives of this study were to (i) determine the temporal and spatial distribution of soil water in the rooting zones of the two neighboring strips during the co-growth period, and (ii) quantify the magnitude of soil water sharing and compensation between the two intercrops under different levels of water availabilities. In a three-year (2009, 2010, 2011) field experiment at the Wuwei Experimental Station of Gansu Agricultural University (37°96′N, 102°64′E), a cool-season pea (*Pisum sativum* L.) crop was ‘relay-planted’ with a warm-season maize (*Zea mays* L.) in alternate field strips (Fig. 1), under three water availability conditions: mild water deficit (30% less than optimal), sub-optimal (15% less than optimal), and optimal (recommended irrigation amounts for the corresponding crops). The three water availability treatments were implemented by controlling irrigation amounts (STable 1). The central hypothesis of the study is that strip-intercropping enhances crop yield and WUE may be related to (i) improved temporal distribution of soil water during the co-growth period, and (ii) the shorter-season pea provides a compensatory effect for soil water to the longer-season maize plants after pea harvest. To test the hypothesis, we designed the field experiment with a layout of the intercrop strips as such that the maize and pea strip-intercropping with no artificial root barrier between the two strips (designated as M/P system) and maize and pea strip-intercropping with a solid plastic sheet inserted between the two strips prior to sowing (designated as PM/P system). In the latter treatment, the plastic sheet inserted between the maize and pea strips enabled a physical block of potential water movement and root penetration from one strip to the other (Fig. 1b). In the former treatment, by contrast, belowground interspecies activities may occur, such as root penetration and water and nutrient movement from one strip to the other.

## Results

### Strip-intercropping enhanced temporal soil water distribution

**Figure 2:**
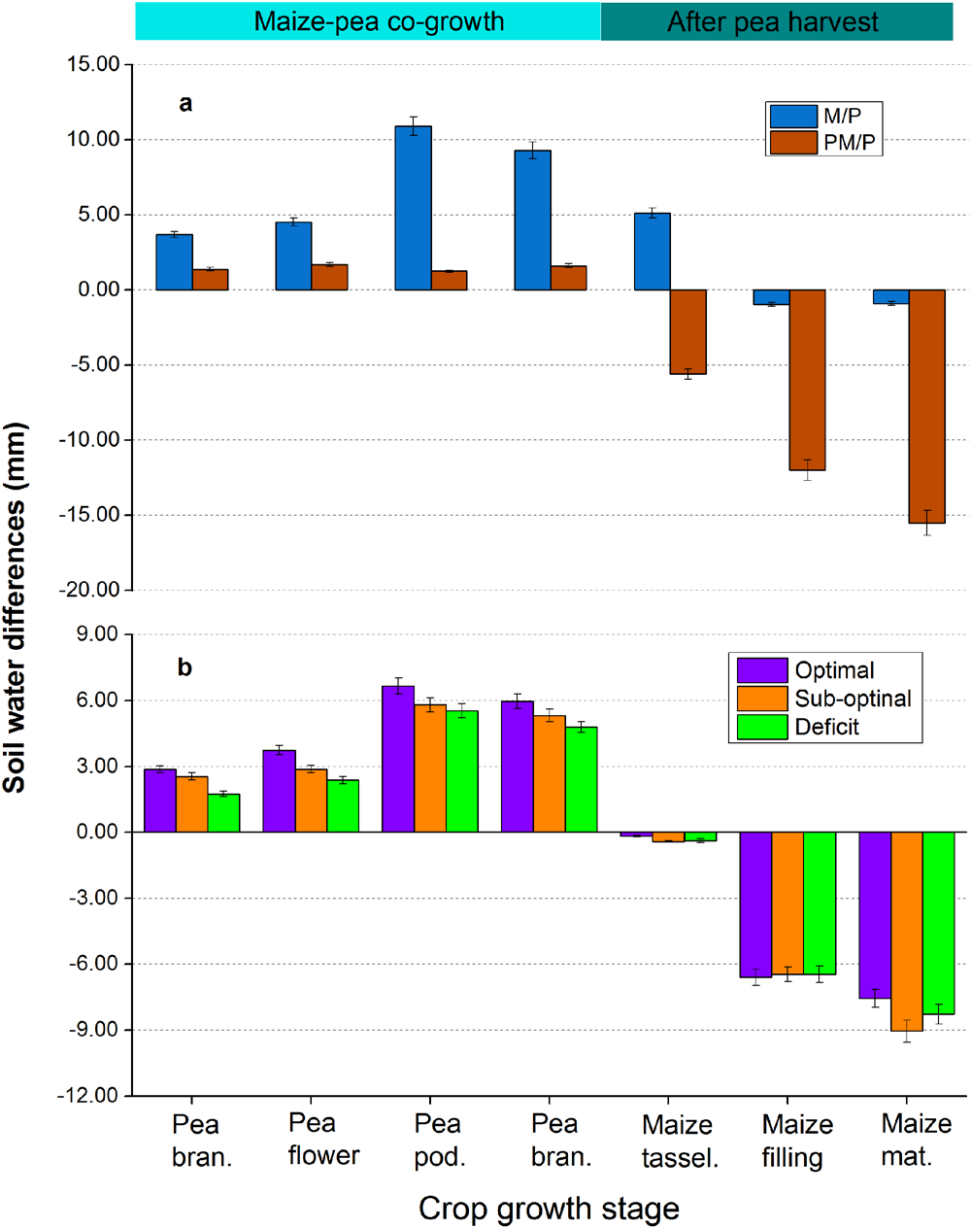
Differences in soil water content between pea and maize strips during the co-growth period and after pea harvest. (**a**) the M/P was compared with PM/P systems, and (**b**) three water availabilities were compared: optimal (recommended irrigation amounts applied to the crops), sub-optimal (15% less than optimal), and deficit (30% less than optimal). Data were three-year means, as treatment effects followed a similar pattern each year. M/P represents the maize-pea strip intercropping with no artificial root barrier between the two crop strips and PM/P represents maize-pea strip intercropping with a solid plastic sheet inserted between the two strips prior to sowing.

In each year, soil water contents were determined in the maize and pea strips during the co-growth period and after pea harvest (Fig. 2**)**. These measurements enabled a quantitative determination of soil water differences between the two strips (ΔSWS) and the temporal change of soil water from one growth stage to the other. We defined that a positive ΔSWS value means that soil water content in the maize strip is greater than that in the pea strip, and vice versa. Results showed that ΔSWS values were all positive during the co-growth period (Fig. 2). On average, the intercropped pea absorbed 23 mm more water from the soil than the intercropped maize during the co-growth period. The cool-season pea was sown about two weeks earlier than the warm-season maize (STable 2); this allowed the early-established pea plants to take the advantage of available soil water before maize plants started their vigorous growth.

After pea harvest, ΔSWS values became negative and the substantial differences were shown between the two systems (Fig. 2). In the M/P system (in which no artificial root barrier was applied between the two strips), ΔSWS values were shifted from an average of 5.1 mm at maize tasseling to a negative value of −1.1 mm at maize harvesting. In the PM/P system (where a solid plastic sheet was inserted between the maize and pea strips), the ΔSWS values shifted from −5.6 mm at maize tasseling to −15.5 mm at maize maturity. These negative values indicated that soil water content in the pea strips were greater than in the maize strips.

The root barrier treatments had a significant effect on ΔSWS. During the co-growth period, the M/P treatment had significantly greater ΔSWS values than the PM/P treatments (Fig. 2a). The accumulated ΔSWS value in the M/P system was averaged 28 mm, significantly greater than ΔSWS value of 5.6 mm in the PM/P system. After pea harvest, the M/P system had a narrow range of ΔSWS value, whereas the PM/P system had significantly greater negative ΔSWS value averaging −33.1 mm.

Soil water availability treatments (via irrigation control, STable 1) had a significant effect on ΔSWS at the different crop growth stages (Fig. 2b). During the co-growth period, the accumulated ΔSWS under the optimal water availability treatment was 16% greater compared to sub-optimal treatment and was 32% greater compared to the water deficit treatment. After pea harvest, all ΔSWS values became negative, with the sub-optimal treatment having the greatest ΔSWS value at maize maturity.

### Strip-intercropping improved spatial soil water distribution across the rooting zones

**Figure 3:**
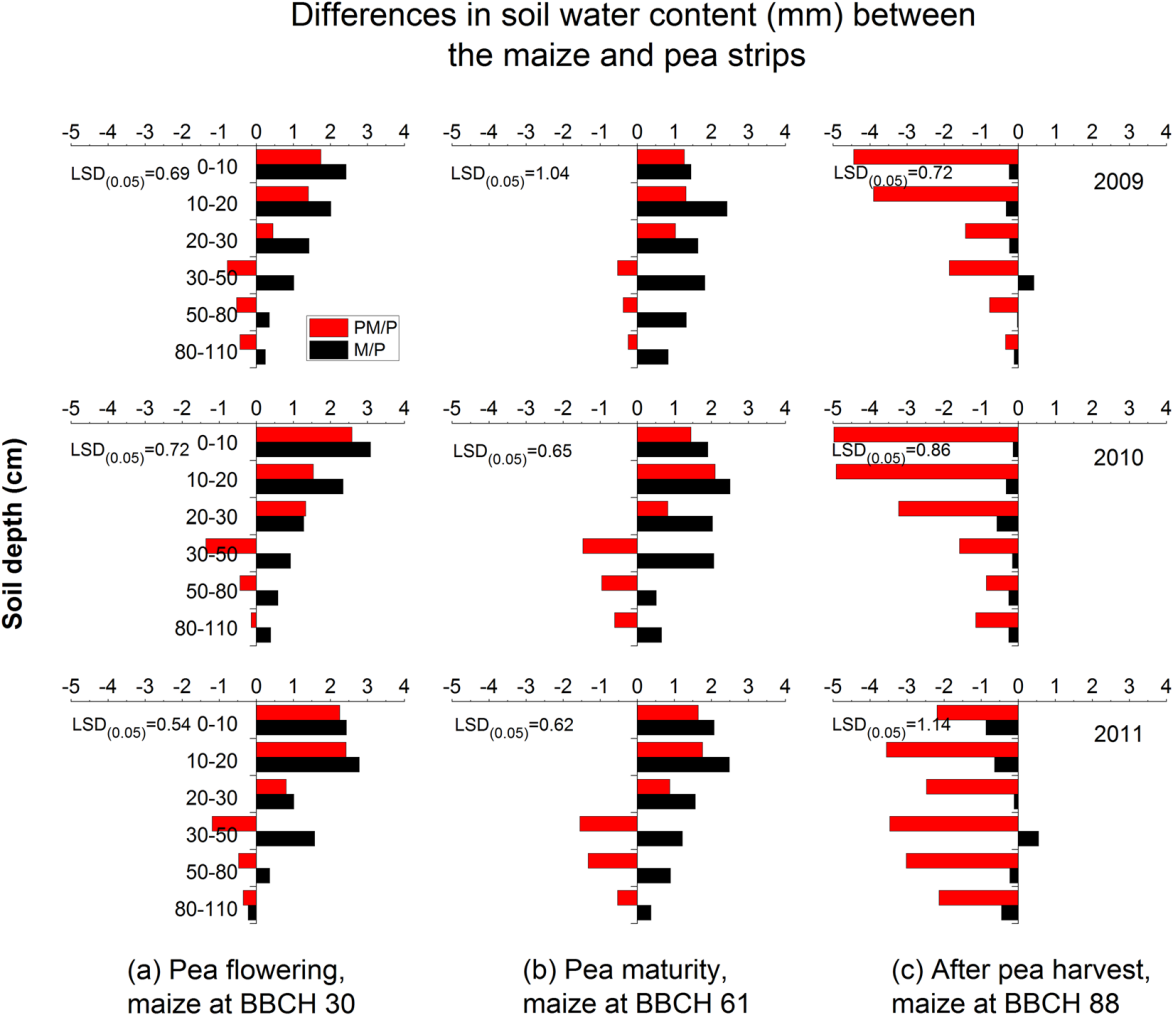
Spatial soil water differences in maize/pea intercropping. The no root barrier (M/P) treatment was compared with the solid plastic sheet barrier (PM/P) treatment at (**a**) pea flowering and maize at stem elongation, (**b**) pea maturity and maize flowering, and (**c**) maize maturity, in 2009, 2010, and 2011. At a given soil depth, positive values indicate that soil water content was higher in the maize strips than in the pea strips, and vice versa. The number in each section is LSD value at *P* < 0.05.

Soil water contents were measured at the three growth stages: (i) pea flowering and maize stem elongation (Fig. 3a), (ii) pea maturity and maize flowering (Fig. 3b), and (iii) maize maturity (Fig. 3c). The ΔSWS values were determined for each soil depth across the 0–110 cm rooting zone. The general trends of the soil water distribution across the 0–110 cm rooting zone were similar in each year, although there were variations among the study years. Overall, the ΔSWS values were mostly positive at each depth at the first two measurement stages (Fig. 3a,b). At a given soil depth, the ΔSWS was greater under the M/P system compared to the PM/P system, indicating that more soil water was absorbed by pea plants than by maize plants. During the co-growth period, soil water in the maize strips of the M/P system was consistently higher in the top three soil layers (0–10, 10–20, and 20–30 cm depths) compared to the pea strips, whereas below the 30-cm depth, soil water contents were generally greater in the pea strips than in the maize strips. These measurements of soil water contents provided an indication of the range of water absorption and the depth of root penetration by the two contrasting crops. The intercropped pea plants extracted soil water mostly from the top 30 cm soil layer, whereas the intercropped maize plants were able to absorb water from the pea strips below the 30-cm layers during the large part of the co-growth periods.

At maize maturity, the ΔSWS values became negative at nearly all the depths (Fig. 3c). Soil water contents in the pea strips became substantially higher compared to the maize strips in the PM/P system. In contrast, soil water contents in the M/P system tended to be balanced between the maize and pea strips. These differences in soil water content between the two intercropping systems suggest that there is spatial variation in water use by the two intercrops.

### Strip-intercropping promoted water sharing via compensatory effect

Solid plastic sheet inserted between the maize and pea strips in the PM/P system prevented soil water from movement between the strips; this contrasted with the M/P system where no physical barrier was artificially applied between the two strips, allowing soil water to exchange freely from one strip to the other. To quantify the amounts of soil water exchanged between the strips, we use two terms to describe the outcomes: water sharing and water compensation. Water sharing was quantified by determining the difference of soil water content between the two strips without root barrier minus the difference in soil water content between the two strips with plastic sheet root barrier (Table 1). This calculation allowed a quantitative assessment of the amount of soil water that maize plants shared with pea plants during the co-growth period. After pea harvest, water movement may have occurred from pea strips to maize strips, providing a potential “compensatory effect” for soil water to the later-maturing maize plants.

**Table 1:** Soil water sharing and compensation between maize and pea strips under different water availabilities.

Water availability	Soil water differences (mm) between the two strips in the M/P system				Soil water differences (mm) between the two strips in the PM/P system				Soil water sharing and compensational effect			
	2009	2010	2011	Mean	2009	2010	2011	Mean	2009	2010	2011	Mean
During the co-growth period									Amounts of water competed between the two strips (mm)^**a**^			
Deficit	32.6a^**c**^	30.6a	28.1a	30.4a	8.9a	8.4a	7.2a	8.2a	23.6a	22.1ab	20.8b	22.2ab
Sub-optimal	29.1a	30.2a	27.3ab	28.8a	4.1b	5.8b	2.9c	4.3b	24.9a	24.4a	24.3a	24.5a
Optimal	23.9b	25.4b	24.8b	24.7b	3.4b	5.3b	4.5b	4.4b	20.5b	20.1b	20.2b	20.3b
During the period after pea harvest									Amounts of water compensated for maize from pea strip (mm)^**b**^			
Deficit	6.3a	5.4a	4.2a	5.3a	−25.9b	−38.2a	−37.0a	−33.7a	32.2b	43.6a	41.3a	39.1a
Sub-optimal	3.9b	2.2b	1.5b	2.5b	−33.1a	−33.5b	−35.7a	−34.1a	37.1a	35.7b	37.4a	36.7ab
Optimal	1.6c	1.2b	1.4b	1.4b	−29.0ab	−35.0ab	−29.8b	−31.3a	30.8b	36.3b	31.3b	32.7b

The soil water sharing was determined during the co-growth period, whereas the water compensation was after pea harvest. The differences in soil water content (mm) were the total amount in the 0–110 cm soil profile, at Wuwei Experimental Station, China, 2009–2010.

^**a**^Water sharing = (differences in soil water content between the two strips in the M/P system)–(differences in soil water content between the two strips in the PM/P system), modified from Chen *et al*.^38^.

^**b**^Soil water compensation for the maize plants from the pea strips after pea harvest.

^**c**^Different letters in the same column in each section denote significant differences at *P* < 0.05.

Water availability had a significant effect on soil water sharing and compensation and the magnitude of the effects varied with year (Table 1). In both 2009 and 2010, soil water sharing under the deficit and sub-optimal water availability treatments was averaged 23.8 mm which was 17% greater than that under optimal water availability treatment. In 2011, however, the water sharing with the sub-optimal treatment was 16% greater compared to the two other water availability treatments. Similarly, water availability had a significant effect on soil water compensation (Table 1). Overall, increasing water availability from deficit to optimal decreased the compensation for soil water in both 2010 and 2011, but this effect was not found in 2009.

### Strip-intercropping increased crop yield and water use efficiency

Root barrier and water availability treatments each had a significant effect on maize yield (Table 2). Averaged across the three water availability treatments, maize grain yield in the M/P system was 27, 26 and 24% higher compared to the PM/P system in 2009, 2010, and 2011, respectively. Pea yield did not differ between the M/P and PM/P systems. The increased total yield led to the land equivalent ratios being 1.33 for the M/P and 1.18 for the PM/P systems, as reported previously^25^.

**Table 2:** Mean grain yields and the ANOVA summary for maize-pea intercropping.

Yield and effect^**a**^	Maize			Pea		
	2009	2010	2011	2009	2010	2011
Mean grain yield (kg ha^−1^)						
Plastic barrier	6736	6989	7539	1662	1716	1737
No barrier	8542	8774	9325	1620	1689	1740
Deficit	6140	6548	6900	1543	1556	1651
Sub-optimal	8240	8421	9026	1666	1760	1765
Optimal	8538	8674	9369	1715	1792	1799
Significance at *P* < 0.05						
Root barrier effect						
*P*-value	0.028	0.021	0.02	0.815	0.719	0.952
LSD (0.05)	665	648	706	172	138	151
Water availability effect						
*P*-value	0.007	0.006	0.006	0.015	0.021	0.036
LSD (0.05)	674	716	765	115	162	108
R × W	NS^**b**^	NS	NS	NS	NS	NS

Two contrasting root barrier treatments were compared under three water availability levels at Wuwei Experimental Station, 2009–2011.

^**a**^No significant interaction was found between root barrier treatment and water availabilities for the traits at the *p* = 0.05 level.

^**b**^NS refers to no significant differences between treatments at 0.05 levels.

Averaged across the two systems, maize grain yield under the optimal and sub-optimal water availabilities were, respectively, 39% and 34% greater compared to deficit treatment in 2009; 32% and 29% greater in 2010; and 36% and 31% greater in 2011. Water availability treatments also had a significant (*p* < *0.05*) effect on the yield of intercropped pea (Table 2). On average, pea yields under optimal and sub-optimal water availability treatments were, respectively, 11% and 8% higher compared to the water deficit treatment in 2009; 15% and 13% higher in 2010; and 7% and 9% higher in 2011.

Root barrier and water availability treatments each had a significant (*p* < 0.05) effect on maize WUE (Table 3). The WUE of maize in the M/P system was 28%, 22%, and 21% greater compared to the PM/P system in 2009, 2010, and 2011, respectively. The WUE of maize under the optimal and sub-optimal water treatments was, respectively, 17% and 23% greater compared to deficit treatments in 2009; 14% and 22% greater in 2010; and 22% and 23% greater in 2011. Unlike intercropped maize, the WUE of the intercropped pea had little response to the root barrier or water availability treatments.

**Table 3:** Mean WUE and the ANOVA summary for maize-pea intercropping.

WUE and effect^**a**^	Maize			Pea		
	2009	2010	2011	2009	2010	2011
Mean WUE (kg ha^−1^ mm^−1^)						
Plastic barrier	10.9	11.7	11.7	5.5	5.7	5.8
No barrier	13.9	14.2	14.1	5.4	5.7	5.8
Deficit	10.9	11.6	11.2	5.4	5.5	5.8
Sub-optimal	13.4	14.1	13.8	5.6	5.9	5.9
Optimal	12.8	13.1	13.7	5.3	5.7	5.7
Significance at *P* < 0.05						
Root barrier effect						
*P*-value	0.018	0.013	0.008	0.834	0.798	0.818
LSD (0.05)	1.4	1.3	1.1	0.4	0.5	0.4
Water availability effect						
*P*-value	0.021	0.011	0.016	0.704	0.601	0.792
LSD (0.05)	1.3	1.5	1.2	0.5	0.6	0.4
R × W	NS^**b**^	NS	NS	NS	NS	NS

Two contrasting root barrier treatments were compared under three water availability levels at Wuwei Experimental Station, 2009–2011.

^**a**^No significant interaction was found between root barrier treatment and water availabilities for the trait at the *p* = 0.05 level.

^**b**^NS refers to no significant differences between treatments at 0.05 levels.

## Discussion

The shortage of fresh water is one of the most severe constraints for agriculture in the arid and semiarid areas^4,26^. Climate change may increase number of extreme weather events in the future^27^, which undoubtedly impacts crop productivity^28,29^. Effective approaches are needed to alleviate the challenge. In the present study, we determined the temporal and spatial distribution of soil water for pea-maize intercropping, and found that soil water content in the pea strips was significantly lower than that in the maize strips during the co-growth period; this was largely due to the superior growth of the intercropped pea over the neighboring maize plants. The cool-season pea was sown about 12 to 18 days earlier than the warm-season maize, providing pea plants with the time-advantages of taking more available water. Also, it might be possible that higher evapotranspiration (not measured) may have occurred in the pea strips.

Pea plants extracted soil water mostly from the shallow (in the top 20 cm) soil depths; this is because the majority of pea roots are concentrated in the 0–30 cm soil profile^30^. By contrast, maize plants have a capacity of rooting to 110 cm in arid and semiarid areas^31,32^. During the co-growth period, intercropped pea only used soil water from the top soil layers, and the unused water in the deeper soil layers may be available to be used by the deeper-rooting intercropped maize. This partly explained why pea-maize intercropping without root barrier (i.e., the M/P system) had a larger soil water difference between the two strips compared to the same intercropping with solid plastic root barrier between the strips (i.e., the PM/P system). The spatial water distribution may be related to the structural differences in rooting between maize and pea plants^33,34^. Asymmetrically constructed rooting systems in strip-intercropping systems have been reported to induce soil water movement from one rooting zone to the other^32,35,36^. The deeper-rooting maize plants might have reached underneath the neighboring shallow-rooting pea strips. Also, it is possible that one intercrop was able to explore a larger soil volume than the other intercrop when the two component crops differ in rooting patterns^37^.

After pea harvest, soil water in the pea strips became freely available either for evaporation or for the progressively growing maize plants to uptake; in the latter case, it would provide a compensatory effect for soil water to the maize plants. Our data indicate a possible interaction belowground that the earlier-maturing pea plants provided some additional soil water to the later-maturing maize plants. This water may have been wasted, otherwise, in a sole pea monoculture system.

Previous studies have suggested that intercropping can be used as a key strategy to reduce runoff and conserve soil moisture^11^ and improve water productivity^38–40^. Our study further demonstrates that intercropping can facilitate the belowground interspecies interactions that promote soil water sharing between the intercrops. A question remains unanswered in the past: how does the level of soil water availability may affect the outcome of soil water sharing? In the present study, we found that water compensation from pea strips to maize strips under the deficit and sub-optimal water availabilities was 7 to 21% greater than that under the optimal treatment. This effect was consistent across the three study years. These results suggest that the outcome of possible soil water sharing between intercrops depends on soil water availability. In practice, the amounts of supplemental irrigation in maize-pea intercropping in arid areas could be decreased by 7 to 21% from the recommended irrigation amount if the interspecies interactions of the two intercrops can be coordinated.

On average, the M/P system increased maize grain yield by an average (three-years) of 25% and enhanced maize WUE by 24% compared to the PM/P system. The mechanisms for the large differences in yield and WUE between the two systems are unknown, but we suggest that the realized enhancements are at least related to three aspects. First, it is related to interspecies water sharing and water compensation. The scenario of water sharing and compensation is supported by the fact that soil water contents in pea strips were significantly higher than in the maize strips when the root barrier (plastic sheet) was inserted between the strips, whereas soil water contents tended to be balanced between the strips when no root barrier was used. Our results provide support to the speculation by previous researchers that the enhanced WUE in intercropping is partly due to improved water sharing and water compensation between the two intercrops^9^.

Second, soil water is the main carrier of nutrients from soil particles to the plant roots. Plants in the two neighboring strips share some of the belowground available nutrients during their co-growth period^22,41^. Belowground interactions between the intercrops are found to promote the rhizosphere processes that stimulate nutrient uptake^42^. Some of the nutrients such as phosphorus can be mobilized by one intercrop to benefit the second intercrop grown in the alternate rows^43^. Intercropping of two crops with contrasting rooting structure can promote nutrient sharing as the root physiological plasticity stimulate belowground interspecific interactions, and enhance root length and lateral root distribution in response to soil nutrients^32^.

Third, in cereal-legume intercropping, root-to-root interactions can led to a significant increase in nodulation and symbiotic N_2_ fixation of the legume^44^. In particular, ameliorating N application in cereal-legume intercropping can improve N_2_-fixation of intercropped legume and increase the complementary growth of intercropped cereal, leading to increased yield of the system^45^. It is also possible that intercrop-induced repellence against pest insects or a stimulation of beneficial soil micro fauna that might contribute to the increased yield^46^. However, there exists important temporal niche differentiation between neighboring intercrops, which provides temporal complementarity for promoting nutrient uptake and increasing nutrient use efficiency^47^.

On a large scope, research on cereal-legume intercropping systems with significantly positive outcomes may have several important implications. For example, the integration of improved farming practices can help increase the systems productivity while reducing the environmental footprints from crop production^48,49^. A more efficient cropping system such as use of intercropping, catch crops^50^, and straw mulching^51^ practices can provide significant benefits to protect the soil from erosion^51^ and improve soil quality^50^, leading to a more stable food production system as described by the United Nations Goals for Sustainability^52^. An efficient cropping system can help maintain a sustainable management of soil resources^51^ and increase soil carbon sequestration^53,54^, and thus enhancing the goods and services that soils can offer to the humankind^55^. In recent years, use of ‘nature-based-solutions’ to enhance the sustainability of cropping systems has attracted great intention. A cereal-legume intercropping, such as the pea-maize intercropping presented in the current study, can potentially serve as a ‘nature-based-solution’ to reduce synthetic chemicals in crop production because the legume in the intercropping can fix N from the atmosphere^56^. A ‘nature-based-solution’ practice uses the theory of system thinking framework to promote desirable soil and landscape functions^57^ and facilitate more rainfall to be conserved in soil by reducing runoff^51^ and thus achieving the sustainability. Relay planting has been identified as a complex suite of different resource-efficient technologies, which possesses the capability to improve soil quality, increase net return, increase land equivalent ratio, and control the weeds and pest infestation^58^. Relay cropping serves as a tool for crop diversification and environmental sustainability with special focus on soil, crop, and long-term sustainability of farming systems.

The climate in northwest China is changing which is having significant impacts on plant phenological development, grain yield, and adaptation strategies^59^. Adopting a single, improved farming practice, such as intercropping^51^, changing seeding date^59^, or shifting sowing date^60^, or use of integrated farming practices^48^ can help alleviate the potential negative impact of changing climate on agriculture and thus the food security. The present study added a significance value to the scientific literature that in the dry areas with high soil evaporation, the increased systems productivity and WUE with intercropping is partly attributable to water sharing through possible water movement between the rooting zones and water compensation from one strip to the other. These water-related effects may lead to other enhancements observed by other researchers, such as the improved rhizosphere processes^42^, enhanced phosphorus mobilization^43^, improved nodulation and symbiotic N_2_ fixation^44^.

## Conclusions

The insertion of the physical barrier (a solid plastic sheet) in the rooting zones between the two intercrops allowed the determination of possible interactions between the two intercrops belowground. We found that certain levels of belowground interspecies interactions occurred when the cool-season, early-sown pea was planted in alternate strips with the warm-season, late-sown maize. Averaged over the three study years, the pea-maize intercropping without a root barrier increased grain yield by 25% and enhanced water use efficiency by 24% compared with the intercropping with the root barrier. Soil water sharing between the intercrops during the co-growth period and water compensation from pea strips to maize strips after pea harvest may have played a role. Other potential belowground mechanisms such as nutrient transfer and soil microbial activities could also contribute significantly to the productivity effects. Using crop species with a contrasting rooting structure for strip intercropping may be able to improve crop productivity through enhancing soil water sharing and water compensation between the intercrops in arid areas.

## Methods

### Experimental site

The field experiment was conducted at the Wuwei Experimental Station of Gansu Agricultural University (37°96′N, 102°64′E, 1506 m a.s.l.), in 2009, 2010, and 2011. Long-term (1960–2010) annual precipitation is 168 mm and annual evaporation is 2400 mm. The average annual sunshine duration is 2945 h, solar radiation is 6000 MJ m^−2^, and the frost-free period is 156 d. In 2009 and 2010, weather conditions were near the long-term averages, whereas in 2011, the growing season precipitation was above the long-term average (SFig. 1). The soil at the experimental site is an Aridisol (serozem), containing total N 0.78 g kg^−1^, Olsen-P 1.41 g kg^−1^ and organic matter 14.3 g kg^−1^.

### Experimental design and treatment implementation

The experiment was arranged in a randomized complete block design with three replicates. In each of the three years, the maize-pea intercropping with no root barrier (i.e., M/P system) was compared with the maize-pea intercropping with solid plastic sheet barrier (i.e., PM/P system), under three water availability conditions: mild water deficit (30% less than optimal), sub-optimal (15% less than optimal), and optimal (recommended irrigation amounts for the corresponding crops); this resulted in 18 experimental units (2 cropping systems × 3 water levels × 3 replicates). Each experimental unit (plot size) was 4.8 × 10 m. A monoculture pea or monoculture maize was not included in the experiment design, because it is well documented in the scientific literature that intercropping has a significant yield advantage than monoculture under various conditions.

For the two maize-pea intercropping systems, two rows of maize (in a 80-cm strip, 40-cm row spacing) were grown in alternate strips with four rows of pea (in a 80-cm strip, 20-cm row spacing), and each plot had three sets of the strips (160 cm per set × 3 sets = 4.8 m in plot width) (Fig. 1). To simplify the presentation, we use ‘M/P’ to represent the maize (M) and pea (P) intercropping with no artificially-applied root barrier between the maize and pea strips (Fig. 1a), and ‘PM/P’ to represent the M/P intercropping that had an artificially-applied root barrier between the maize and pea strips (Fig. 1b). The M/P planting without a physical barrier between the strips allows soil water to move freely in rooting zones, whereas in the PM/P system, the plastic sheet barrier inserted between the maize and pea strips prevented soil water from exchange between the two strips. At each plot, a trench (10 m long × 0.2 m wide × 1.1 m deep) was made, the plastic sheet (1.1 m in depth × 10 m in length × 0.12 mm in thickness) was placed vertically into the middle of the trench, and the trench was filled up with the original soil and packed. The plastic sheet barrier treatment was implemented for each of the treatment plot in each replicate. The length of the trench for the plastic sheet was in the full plot length (10 m).

Seeding rate was 82,500 plants ha^−1^ for maize (cv. Jixiang NO.1) and 240,000 plants ha^−1^ for pea (cv. MZ-1). The phenological growth stages were recorded (STable 2). All plots received 300 kg N ha^−1^ of ammonium nitrate and 90 kg P ha^−1^ as monoammonium phosphate; this fertilizer program was to target an average maize yield of 7.5 t ha^−1^ and pea yield of 1.5 t ha^−1^. Half of the N and all of the P fertilizers were incorporated into the soil prior to sowing, and the remaining half of N fertilizer was divided into two portions, with one being applied at the maize elongation stage (BBCH = 33) and the other at the pre-tasseling stage (BBCH = 55). PVC pipes with diameter of 15 cm were used for plot irrigation. The volume of water applied to the individual plots varied with the treatment design (STable 1). A flow meter was installed at the recharging end to measure and record the amount of irrigation flowing into each plot.

### Measurements

#### Soil water

Soil water content was measured to the depth of 110 cm on each measurement date prior to the irrigation events (STable 1). Soil water contents in the 0–10, 10–20, 20–30 cm depth were measured using the oven-drying method, whereas those in the 30–60, 60–90, and 90–110 cm depths were measured using neutron probe (NMM, model CPN 503 DR, Campbell Pacific Nuclear International Inc., USA) that were installed in each plot prior to sowing (Fig. 1a). Volumetric water content (cm^3^ cm^−3^) was obtained by multiplying the gravimetric water content by soil bulk densities of 1.4, 1.42, 1.47, 1.54, 1.52, and 1.50 g cm^−3^ for the six soil depths. Bulk density was determined from undisturbed soil cores taken at sowing. Volumetric water content was converted into mm using the depth of the soil as follows:

1 $$SWS={\theta }_{v}\times h\times 10$$

where *θ*_*v*_ is the volumetric water content at a specific soil layer (cm^3^ cm^−3^), and *h* is the soil depth increment (cm).

The differences in soil water content between the two intercrop strips (ΔSWS) was defined as the soil water content in the maize strips minus that in the pea strips at a specific soil depth, as follows:

2 $${\rm{\Delta }}SWS=SW{S}_{Maize}-SW{S}_{Pea}$$

A positive value indicates that maize strips had a greater soil water storage than pea strips at the given soil depth. ΔSWS was calculated for the period from one irrigation event to the next during the entire growing season.

#### Water sharing and compensation

Water sharing was defined as the amount of soil water absorbed by one intercrop from the other intercrop during their co-growth periods^38^. Thus, water sharing (ΔΔ*SWS*_*Co-*_, mm) was calculated as the difference in soil water content between the maize and pea strips without root barrier (Δ*SWS*_(M/P)_) minus the difference in soil water content between the two strips with plastic root barrier (Δ*SWS*_(PM/P)_), as follows:

3 $${\rm{Water}}\,{\rm{competition}}\,({\rm{\Delta }}{\rm{\Delta }}SW{S}_{Co-})={\rm{\Delta }}SW{S}_{({\rm{M}}/{\rm{P}})}-{\rm{\Delta }}SW{S}_{(\mathrm{PM}/{\rm{P}})}$$

Similarly, water compensation (ΔΔ*SWS*_*After-*_, mm) was defined as the amount of soil water absorbed by the late-maturing maize plants from the early-maturing pea strips during the period after the pea harvested, as follows:

4 $${\rm{Water}}\,{\rm{compensation}}\,({\rm{\Delta }}{\rm{\Delta }}SW{S}_{After-})={\rm{\Delta }}SW{S}_{({\rm{M}}/{\rm{P}})}-{\rm{\Delta }}SW{S}_{(\mathrm{PM}/{\rm{P}})}$$

where Δ*SWS*_(M/P)_ was the difference in soil water content between the two crop strips without root barrier, and Δ*SWS*_(PM/P)_ was the difference in soil water content between the two strips with plastic root barrier, both being measured after pea harvest.

#### Grain yield and WUE

All plants in each plot were hand-harvested at full maturity. The cobs were threshed, air-dried, cleaned, and weighed. Grain yield was reported on a dry weight basis. Seasonal evapotranspiration (ET_c_ in mm) was determined using water balance equation as follows:

5 $${\rm{ET}}=P+I+U-R-{D}_{w}-{\Delta }S$$

where *P* is the rainfall (mm) during the growing seasonal (from sowing to harvest) for each crop, *I* the irrigation (mm), *U* the upward capillary flow into the root zone (mm), *R* the runoff (mm), *D*_*w*_ the downward capillary flow into the root zone (mm), and *ΔS* is the change of soil water stored in the 0–110 cm soil layer. The upward and downward flows were measured previously^61^ at a nearby site with environmental conditions nearly identical to those at our experimental site. These authors indicated that the two items were negligible in this semiarid area. Runoff was also negligible due to small rains, and irrigation was controlled by raised ridges between plots. The *ΔS* value was calculated as the difference of soil water content at sowing and at crop physiological maturity.

Water use efficiency (WUE) was calculated using the following formula:

6 $${\rm{WUE}}={\rm{Y}}/{\rm{ET}}$$

where Y is the grain yield of maize and pea (kg ha^−1^), ET is the total actual evapotranspiration over the whole growing season calculated from Eq. (5). The summed yield of pea and maize was used in the WUE determination, giving an indication of the WUE for the whole intercropping system, rather than each of the two individual crops included in the system. We realize that pea and maize grains have a totally different energy synthesis costs and these energy costs should be considered in future studies to determine the effect of cropping systems on energy use efficiency.

### Statistical analysis

Analysis of variance was performed on all measured variables using statistical package of SPSS (SPSS software, 17.0, SPSS Institute Inc., USA) with the standard factorial RCBD analysis method. Cropping systems (C), soil water availability treatments (W), and their interactions (C x W) were considered the fixed effect and replicates the random effect in ANOVA. When C x W interaction was not significant, the mean effect was presented; however, when C x W interaction was significant, the water availability effect was determined for each of the two intercropping systems, and system effect was determined for each of the three water availability treatments. When year by treatment interaction was significant, the treatment effect was discussed for each year, and otherwise, three-year averages were presented. Significant treatment effects were presented at the *P* < 0.05 level.

### Data availability

The datasets generated during and/or analysed during the current study are available from the corresponding author on reasonable request.

## Electronic supplementary material

Supplementary Information
