# Supplementary material for: Enhancing the systems productivity and water use efficiency through coordinated soil water sharing and compensation in strip-intercropping
Source: Sci Rep. 2018 Jul 12;8:10494. doi: 10.1038/s41598-018-28612-6 (PMC6043509; doi:10.1038/s41598-018-28612-6)
Supplement: Supplementary file 1 — Supplementary Information [file 41598_2018_28612_MOESM1_ESM.doc]

**Supplementary Information**

**Enhancing the systems productivity and water use efficiency through coordinated soil water sharing and compensation in strip-intercropping**

Guodong Chen^1,3^, Xuefu Kong^1,2^, Yantai Gan^4^, Renzhi Zhang^1^, Fuxue Feng^1^, Aizhong Yu^1,2^, Cai Zhao^1^, Sumei Wan^3^, Qiang Chai^1,2*^

**a**

**b**

**c**

**Supplementary Figure 1.** Weather conditions during the three study years with (**a**) daily mean air temperature, (**b**) monthly precipitations, and (**c**) monthly evapotranspiration. The inverted green triangle points in (b) indicate the long-term (1961-2010) average monthly precipitation at the experimental site.

| **Supplementary Table 1.** The amount of irrigation applied to the crops at the different growth stages, at Wuwei Experimental Station, northwestern China, 2009-2011 | | | | | | | | | | |
| --- | --- | --- | --- | --- | --- | --- | --- | --- | --- | --- |
| Treatment **^a^** | Pea branching | Pea flowering | Pea filling | Maize pre-heading | Maize flowering | Maize filling |  | Total | | |
|  |  |  |  |  |  |  |  | 2009 | 2010 | 2011**^b^** |
|  |  mm  | | | | | | | | | |
| M/P1 | 72 | 72 | 72 | 72 | 72 | 60 |  | 420 | 420 | 360 |
| M/P2 | 80 | 80 | 80 | 80 | 80 | 70 |  | 470 | 470 | 400 |
| M/P3 | 90 | 90 | 90 | 90 | 90 | 75 |  | 525 | 525 | 450 |
| PM/P1 | 72 | 72 | 72 | 72 | 72 | 60 |  | 420 | 420 | 360 |
| PM/P2 | 80 | 80 | 80 | 80 | 80 | 70 |  | 470 | 470 | 400 |
| PM/P3 | 90 | 90 | 90 | 90 | 90 | 75 |  | 525 | 525 | 450 |
| **^a^** M/P, maize-pea intercropping with no root barrier between intercropped maize and intercropped pea strips; PM/P, maize-pea intercropping with plastic sheet barrier inserted between the two intercrop strips at sowing; 1, 2, and 3 means the three irrigation levels, representing water deficit (30% less than optimal), sub-optimal (15% less than optimal), and optimal (recommended irrigation amount), respectively. For example, PM/P2 means maize-pea intercropping with plastic sheet barrier under sub-optimal water condition; | | | | | | | | | | |
| **^b^** In 2011, no irrigation was applied at maize grain filling stage due to a large rainfall. | | | | | | | | | | |

| **Supplementary Table 2.** Phenological stages of pea and maize plants (BBCH scale) at Wuwei Experimental Station, 2009-2011. | | | | | | | |
| --- | --- | --- | --- | --- | --- | --- | --- |
| Year | Crop | Sowing | Seedling  (BBCH 13) | Branching/jointing  (BBCH 55) | Flowering /tasseling  (BBCH 65) | Podding/filling  (BBCH 75) | Maturity  (BBCH 92) |
| 2009 | Maize | 19-Apr **^a^** | 8-May | 16-June | 15-July | 12-Aug | 24-Sep |
|  | Pea | 6-Apr | 19-Apr | 7-May | 21-May | 3-June | 6-July |
|  |  |  |  |  |  |  |  |
| 2010 | Maize | 22-Apr | 12-May | 18-June | 16-July | 12-Aug | 26-Sep |
|  | Pea | 6-Apr | 22-Apr | 7-May | 21-May | 6-June | 8-July |
|  |  |  |  |  |  |  |  |
| 2011 | Maize | 20-Apr | 7-May | 16-June | 19-July | 14-Aug | 28-Sep |
|  | Pea | 8-Apr | 23-Apr | 7-May | 21-May | 5-June | 6-July |
| **^a^** The date was recorded when 75% of the plants were developed to the particular growth stage in a plot. Irrigation treatments did not cause differences in plant growth stages. | | | | | | | |
